# Simultaneous Classification of Objects with Unknown Rejection (SCOUR) Using Infra-Red Sensor Imagery

**DOI:** 10.3390/s25020492

**Published:** 2025-01-16

**Authors:** Adam Cuellar, Daniel Brignac, Abhijit Mahalanobis, Wasfy Mikhael

**Affiliations:** 1 Center for Research in Computer Vision, University of Central Florida, Orlando, FL 32816-8005, USA; 2 Department of Electrical and Computer Engineering, University of Arizona, Tucson, AZ 85721-0104, USA; dbrignac@arizona.edu (D.B.); amahalan@arizona.edu (A.M.); 3 Department of Electrical and Computer Engineering, University of Central Florida, Orlando, FL 32816-8005, USA; wasfy.mikhael@ucf.edu

**Keywords:** infra-red, ATR, target classification, unknown rejection, OOD, open-set recognition

## Abstract

Recognizing targets in infra-red images is an important problem for defense and security applications. A deployed network must not only recognize the known classes, but it must also reject any new or *unknown* objects without confusing them to be one of the known classes. Our goal is to enhance the ability of existing (or pretrained) classifiers to detect and reject unknown classes. Specifically, we do not alter the training strategy of the main classifier so that its performance on known classes remains unchanged. Instead, we introduce a second network (trained using regression) that uses the decision of the primary classifier to produce a class conditional score that indicates whether an input object is indeed a known object. This is performed in a Bayesian framework where the classification confidence of the primary network is combined with the class-conditional score of the secondary network to accurately separate the unknown objects from the known target classes. Most importantly, our method does not require any examples of OOD imagery to be used for training the second network. For illustrative purposes, we demonstrate the effectiveness of the proposed method using the CIFAR-10 dataset. Ultimately, our goal is to classify known targets in infra-red images while improving the ability to reject unknown classes. Towards this end, we train and test our method on a public domain medium-wave infra-red (MWIR) dataset provided by the US Army for the development of automatic target recognition (ATR) algorithms. The results of this experiment show that the proposed method outperforms other state-of-the-art methods in rejecting the unknown target types while accurately classifying the known ones.

## 1. Introduction

Recognizing targets in infra-red images is an important problem for defense and security applications. In part, the challenge is that a deployed network must not only recognize the known classes, but it must also reject any new or unknown objects without confusing them to be one of the known classes [1,2]. This is also known as the problem of dealing with “out-of-distribution” (OOD) data or as the “open-set recognition” problem [3,4].

Since the number of unknown object types is immeasurable, it is not possible to exhaustively train the classifier to handle OOD images. One of the simplest strategies is to use a threshold for rejecting low confidence decisions as unknown objects. In fact, it has been observed that a strong classifier (with high correct classification accuracies) is able to effectively reject OOD data using a decision confidence threshold [5]. Other methods utilize a limited number of OOD images or generate virtual outlier categories, as in the case of the MOS framework, which introduces an ‘others’ category into each semantic group to aid in rejecting OOD data without external datasets, and generally benefits the rejection of other OOD data [6,7]. It has also been demonstrated that contrastive learning and the use of metrics to form tight clusters enhance a network’s ability to separate known and unknown classes [8]. Additionally, the introduction of placeholders has been shown to improve model calibration for open-set recognition [9].

Unlike most other methods, our goal is to enhance the ability of existing (or pretrained) classifiers to detect and reject unknown classes. Specifically, we do not alter the training strategy of the main classifier so that its performance on known classes remains unchanged. Instead, we introduce a second network that uses the decision of the primary classifier to produce a class-conditional score for whether the input object is indeed a known object. This is achieved using a Bayesian framework [10], where the classification confidence of the primary network is combined with the class-conditional score of the secondary network to accurately separate unknown objects from known target classes. Most importantly, our method does not require any examples of OOD imagery, and outperforms most other strategies reported in the literature.

Ultimately, our goal is to classify known targets in infra-red images while improving the ability to reject unknown classes. To this end, we train and test our method on a public domain medium-wave infra-red (MWIR) dataset provided by the US Army for the development of automatic target recognition (ATR) algorithms [11]. For illustrative purposes, we also demonstrate the effectiveness of the proposed method using the CIFAR-10 dataset. Section 2 is a review of some relevant papers in the literature. The proposed method and the Bayesian framework in which it is developed are described in Section 3. The details of the datasets and experiments are described in Section 4. Section 5 provides a summary along with the main conclusions of the paper.

## 2. Background Review

Miller et al. introduce two significant concepts for handling the OOD problem (which is also known as open-set recognition). First, they propose class anchor clustering (CAC) loss, which encourages the formation of tight, class-specific clusters in the feature space. These tightly bound clusters enhance the model’s ability to distinguish between known and unknown classes at deployment. The CAC loss comprises two parts: (1) a modified Tuplet loss, which pushes data points away from incorrect class centers while pulling them toward the correct class center; and (2) an anchor loss, which penalizes the distance between a data point and its correct class center, further solidifying class clusters. Second, Miller et al. use anchored class centers in the logit space, fixing the positions of class centers during training to ensure stable, well-separated clusters for better open-set detection [8].

Chen et al. expand on the reciprocal points learning (RPL) framework by introducing adversarial reciprocal points learning (ARPL). In this method, each class is represented by a reciprocal point in the feature space, where the reciprocal point encapsulates the “otherness” of the class. The probability that a sample belongs to a known class is proportional to its distance from these reciprocal points, with the assumption that unknown (open set) examples will be distant from all known class points. ARPL enhances this by computing the feature distances using both Euclidean and cosine distances, providing a more robust separation of known and unknown classes. The method is further strengthened with ARPL + confusing samples (ARPL + CS), which adversarially generates synthetic points (confusing samples) that represent potential unseen class instances. These confusing samples are constrained to be equidistant from all reciprocal points, maintaining consistency with ARPL’s open-set scoring rule [12].

Vaze et al. build upon the foundational work in open-set recognition introduced by Scheirer et al., demonstrating a strong correlation between a model’s closed-set and open-set performance. They leverage this insight to improve their maximum softmax probability (MSP) baseline, a widely used technique for detecting out-of-distribution samples. By incorporating advanced techniques from the image recognition field, such as extended training durations, enhanced data augmentations, and label smoothing, they significantly boost MSP’s performance. Notably, these optimizations allow them to surpass even advanced methods like ARPL + CS, highlighting the critical role of training strategies in open-set recognition’s success [5].

## 3. Proposed Approach

**Figure 1:**
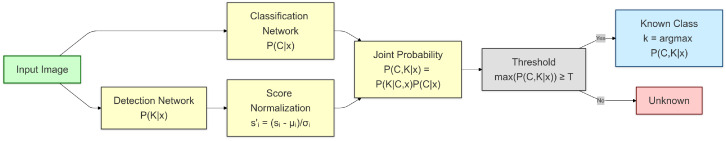
Overview of the proposed SCOUR framework for simultaneous object classification and unknown rejection.

We propose an approach for open-set recognition in infra-red imagery using two neural networks: a classifier network and a single detector network. The classifier network is trained to recognize known classes, assigning probabilities to each class for a given input image. The detector network is responsible for distinguishing between in-distribution (known) and out-of-distribution (unknown) inputs, learning representations that generalize across all classes. An overview of the approach is represented in Figure 1.

The goal is to model the joint probability that an input image belongs to a known class and whether the input is from a known or unknown class. Specifically, we want to estimate the joint probability P(C,K∣x) or P(C,U∣x), where *C* represents the class label assigned by the classifier, and *K* and *U* represent the likelihood that the input is from a known or unknown class, respectively. This joint probability is calculated as follows:

(1) P(C,K∣x)=P(K∣C,x)P(C∣x),

(2) P(C,U∣x)=P(U∣C,x)P(C∣x),

where P(C∣x) is the classifier network’s output representing the probability that the input belongs to class *C*, and P(K∣C,x) and P(U∣C,x) are the probabilities that the input is known or unknown. The relationship between P(K∣C,x) and P(U∣C,x) is given as follows:

(3) P(K∣C,x)=1−P(U∣C,x).

This allows us to model both the classification decision and the uncertainty about whether the input is from a known or unknown distribution by multiplying the classifier’s output with the detector’s output.

Our approach utilizes a single detection network that operates across all classes. This network produces a probability estimate, P(K∣x) or P(U∣x), determining whether the input is likely known. These probabilities are combined with the classifier’s output P(C∣x) to compute the joint probability P(C,K∣x).

After training both networks, we normalize the outputs of the detector network for each class. Specifically, for each class *i*, we compute the mean $\mu_{i}$ and standard deviation $\sigma_{i}$ of the detector network’s outputs:

(4) $$\mu_{i}=\frac{1}{N_{i}}\sum_{j=1}^{N_{i}}s_{i}^{\left(j\right)},\;\sigma_{i}=\sqrt{\frac{1}{N_{i}}\sum_{j=1}^{N_{i}}{\left(s_{i}^{\left(j\right)}-\mu_{i}\right)}^{2}},$$

where $s_{i}^{\left(j\right)}$ is the detector network’s output for sample *j* of class *i*, and $N_{i}$ is the number of validation samples for class *i*. We then normalize the detector outputs for each class:

(5) $$s_{i}^{\prime }=\frac{s_{i}-\mu_{i}}{\sigma_{i}},$$

where $s_{i}$ is the detector output for a given input, and $s_{i}^{\prime }$ is the normalized score for class *i*. This process converts raw scores to the *t*-statistic. The key property of the *t*-statistic is that it is a pivotal quantity—while defined in terms of the sample mean, its sampling distribution does not depend on the population parameters, and thus it can be used regardless of what these may be [13]. To integrate the outputs from both networks, we perform element-wise multiplication of the normalized detector scores with the classifier’s probability scores:

(6) $$P_{\mathrm{final}}=P_{\mathrm{class}}⊙s^{\prime },$$

where $s^{\prime }=\left[s_{1}^{\prime },s_{2}^{\prime },\ldots ,s_{C}^{\prime }\right]$ is the vector of normalized detector scores for all *C* known classes. Finally, we apply a threshold *T* to the combined scores to determine if an input image belongs to a known or unknown class:

(7) $$\left\{\begin{array}{cc} \mathrm{Assign}\;\mathrm{to}\;\mathrm{class}\;k=\arg\max(P_{\mathrm{final}}), & \mathrm{if}\;\max(P_{\mathrm{final}})\geq T,\\ \mathrm{Classify}\;\mathrm{as}\;\mathrm{unknown}, & \mathrm{if}\;\max(P_{\mathrm{final}})<T. \end{array}\right.$$

The threshold *T* is an operational parameter that allows the end user to adjust the trade-off between the probability of detecting known objects and the probability of false alarms when encountering unknown objects. The specific value of *T* will depend on the requirements and risk tolerance of the particular application.

By combining the classifier network with the single detection network, our method improves open-set recognition performance. This approach effectively models both the classification decision and the uncertainty regarding whether the input is from a known or unknown class using a joint probability framework.

## 4. Experiments

In this section, we describe the datasets used, the experimental setup, and the training procedures for evaluating our proposed approach.

### 4.1. Datasets

We evaluated our method on two datasets: CIFAR-10 and the Defense Systems Information Analysis Center (DSIAC) infra-red imagery dataset.

#### 4.1.1. CIFAR-10

The CIFAR-10 dataset consists of 60,000 color images of size 32×32 pixels, divided into 10 classes with 6000 images per class. The classes are: airplane, automobile, bird, cat, deer, dog, frog, horse, ship, and truck. The dataset is split into 50,000 training images and 10,000 test images.

For our experiments, we focused on distinguishing between animal and non-animal classes. We considered the six animal classes as known classes: bird, cat, deer, dog, frog, and horse. The four non-animal classes—airplane, automobile, ship, and truck—were treated as unknown during testing.

We removed all images belonging to the unknown classes from the training set. Thus, the classifier and detector networks were trained using only the images from the known animal classes. During testing, we used images from both the known animal classes to evaluate classification performance and images from the unknown non-animal classes to assess the open-set recognition capability.

#### 4.1.2. DSIAC

**Figure 2:**
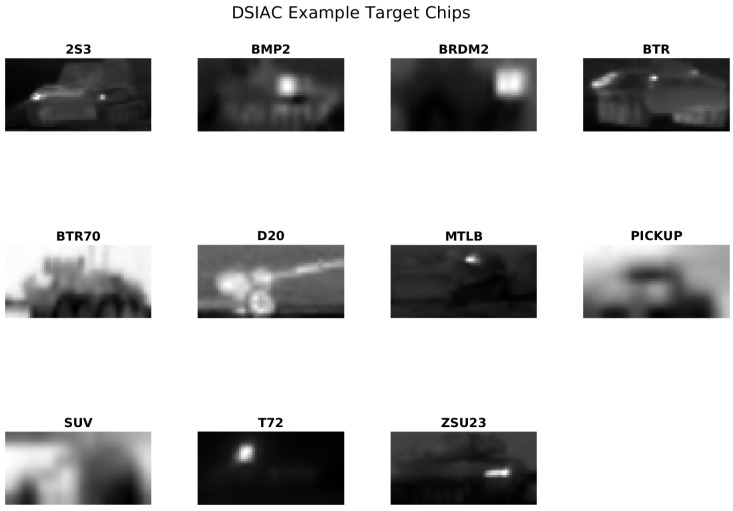
Example target chips from the DSIAC dataset. The images represent different vehicle classes, including both military and civilian vehicles.

The DSIAC dataset is a dataset containing various military and civilian vehicle classes captured under different conditions. It consists of 11 classes: 2S3, BMP2, BRDM2, BTR, BTR70, D20, MTLB, PICKUP, SUV, T72, and ZSU23. Two primary sensors were used to collect the target imagery, one operating in the MWIR portion of the spectrum and one operating in the visible band. Each sensor was assigned a four-character identifier that is used in the file naming scheme—“cegr” is the NVESD nomenclature for the L3 Cincinnati Electronics Night Conqueror MWIR imager that was combined with a Great River frame grabber to extract data. The Night Conqueror camera uses a 640 × 480 pixel Indium Antimonide (InSb) focal plane array (FPA) with a 28-micron pitch. The system used a fixed FOV 300 mm lens resulting in a 3.4 × 2.6 FOV and had a CO2 notch cold filter installed. The visible light imagery was collected using a camera manufactured by Illunis that was referred to as “i1co” in the NVESD nomenclature. A Nikon zoom lens was adjusted to produce a 3.4-degree HFOV and locked-in position. The output imagery was collected using a Coreco framegrabber [14]. For our experiments, we use only the MWIR imagery. Example images are shown in Figure 2.

For our initial evaluation, we established a baseline experiment using a straightforward split of the data.

#### 4.1.3. DSIAC Initial Experiment

In our initial experiment, we selected the first seven classes as known: 2S3, BMP2, BRDM2, BTR, BTR70, D20, and MTLB. The remaining four classes—PICKUP, SUV, T72, and ZSU23—were considered unknown.

We removed all images of the unknown classes from the training set. The classifier and detector networks were trained using images from the known classes only. During testing, we included images from both the known and unknown classes to evaluate the performance of our method in recognizing known classes and detecting unknown inputs.

To further assess the robustness of our method, particularly in challenging scenarios, we conducted additional experiments using a more difficult data split.

#### 4.1.4. DSIAC Tracked vs. Wheeled

In this experiment, we aimed to classify tracked vehicles while identifying wheeled vehicles as unknown. The known classes were the tracked vehicles: 2S3, BMP2, MTLB, T72, and ZSU23. The unknown classes were the wheeled vehicles: BRDM2, BTR, BTR70, D20, PICKUP, and SUV. The classifier and detector networks were trained using only images from known tracked vehicle classes.

#### 4.1.5. DSIAC Wheeled vs. Tracked

In this experiment, the objective was to classify wheeled vehicles and identify tracked vehicles as unknown. The known classes consisted of the wheeled vehicles: BRDM2, BTR, BTR70, D20, PICKUP, and SUV. The unknown classes were the tracked vehicles: 2S3, BMP2, MTLB, T72, and ZSU23. The networks were trained exclusively on the wheeled vehicle images.

### 4.2. Training Procedure

We conducted experiments for both datasets to evaluate the effectiveness of our proposed approach. We used two network architectures: the Classifier32 network, which is the same architecture employed by ARPL + CS and the Good Classifier baseline, and a ResNet-18 network to investigate whether a residual architecture could further improve performance [15].

In our experiments using the ResNet-18 network, we made specific modifications to the original architecture to better accommodate the small input image size of 32 × 32 pixels used in the CIFAR-10 and resized DSIAC datasets. Specifically, we adjusted the first convolutional layer to have a kernel size of 3 × 3 with a stride of 1 and padding of 1, replacing the original 7 × 7 kernel with a stride of 2 and padding of 3. This modification reduces the initial downsampling, preserving more spatial details from the input images. Additionally, we removed the max-pooling layer that typically follows the first convolutional layer in ResNet-18. These changes help maintain higher spatial resolution in the feature maps throughout the network, which is beneficial when working with small images like those in our datasets.

For both network architectures, we trained two neural networks independently:

- Classifier Network: Trained using labeled images from the known classes to perform multi-class classification.
- Detector Network: Configured similarly and trained using the same images but with class-specific target vectors (e.g., one-hot encoded vectors) to learn distinct representations for each class. The network minimizes the mean squared error between its outputs and the target vectors.

The images were preprocessed by normalizing pixel values. For CIFAR-10, images were used at their original size of 32×32 pixels. For the DSIAC dataset, images were resized to 32×32 pixels to match the input size required by our networks.

Training was performed using the AdamW optimizer [16] with an initial learning rate of $1\times 10^{-3}$. Hyperparameters were chosen based on the performance observed on a validation set. For the Classifier32 experiments, we trained the networks for up to 100 epochs on CIFAR-10 and 120 epochs on DSIAC. For the ResNet-18 experiments, the networks were trained for up to 40 epochs on CIFAR-10 and 40 epochs on DSIAC.

### 4.3. Evaluation

We evaluated the models on the test sets of each dataset. For the known classes, we measured classification accuracy. For the unknown classes, we assessed the open-set recognition performance by constructing receiver operating characteristic (ROC) curves and computing the area under the curve (AUC).

The ROC curve was constructed by varying a threshold *T* over the range of combined scores. For each threshold value, we computed the following:

- Probability of Detection (PD): The proportion of known class samples where ${\mathrm{Score}}_{\mathrm{known}}>T$.
- Probability of False Alarm (PFA): The proportion of unknown class samples where ${\mathrm{Score}}_{\mathrm{unknown}}>T$.

By plotting PD (y-axis) against PFA (x-axis) for various threshold values *T*, we obtained the ROC curve. The y-axis represents the probability of correctly identifying known samples as known, while the x-axis represents the probability of incorrectly identifying unknown samples as known.

This approach directly measures the trade-off between correctly detecting known samples and falsely accepting unknown samples as known, which is crucial in open-set recognition tasks.

We observed that our method achieved high AUC values on both datasets, indicating effective discrimination between known and unknown classes. By selecting an appropriate threshold *T*, one can balance the detection rate of known classes with the false alarm rate for unknown classes, depending on the requirements of the application.

In addition to ROC curves, we also calculated the classification accuracy on the known classes to assess the performance of the classifier network independently. The combined use of both networks and the score normalization improved open-set recognition performance without compromising classification accuracy on known classes.

We conducted the initial experiments using two different network architectures: the Classifier32 network and the ResNet-18 network. We compare our results with the methods mentioned in Section 2, including ARPL + CS, CAC, and the Good Classifier baseline.

**Figure 3:**
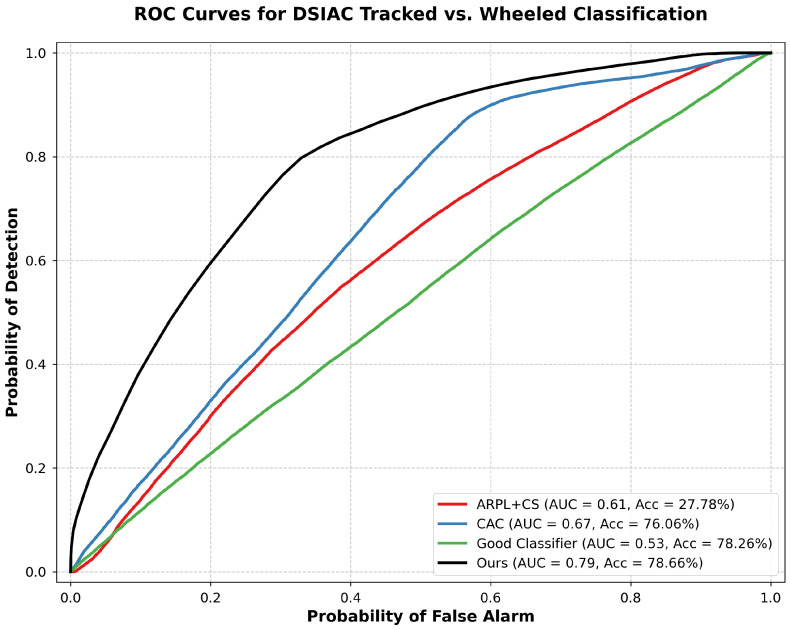
ROC curves for open-set recognition on the DSIAC dataset where tracked vehicles are known and wheeled vehicles are unknown, comparing our approach with ARPL + CS, CAC, and Good Classifier.

**Figure 4:**
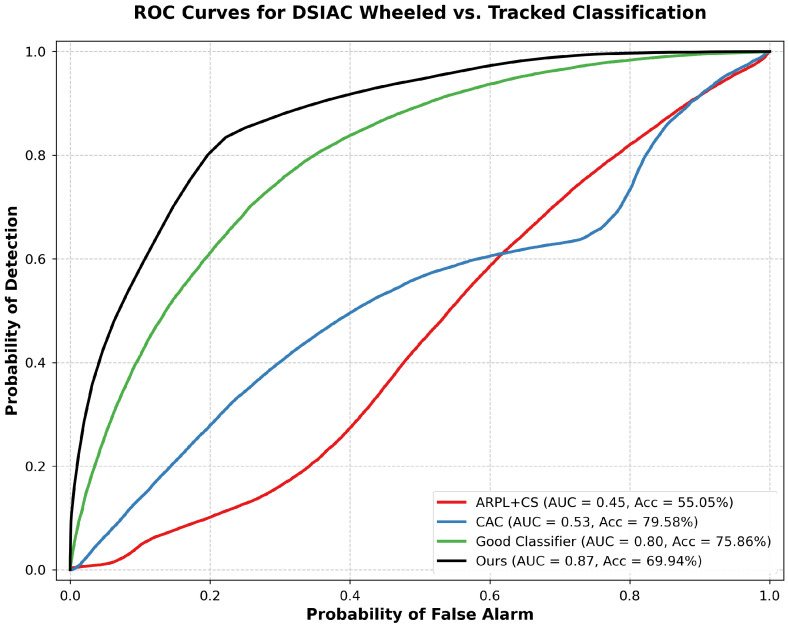
ROC curves for open-set recognition on the DSIAC dataset where wheeled vehicles are known and tracked vehicles are unknown, comparing our approach with ARPL + CS, CAC, and Good Classifier.

Table 1 summarizes the performance comparison across the initial DSIAC experiment and the CIFAR-10 dataset for both network architectures. The results for the additional DSIAC experiments, using the Classifier32 network, are shown in Figure 3 and Figure 4.

For the CIFAR-10 dataset, using the Classifier32 network, our approach achieved a classification accuracy of 88.18% and an AUC of 0.8654 in open-set recognition. This demonstrates the effectiveness of our method in accurately classifying known classes while effectively detecting unknown inputs, ensuring a fair comparison with existing methods that use the same architecture.

When using the ResNet-18 network, we observed an improvement in classification accuracy to 92.47%, although the AUC decreased slightly to 0.8294. This suggests that while a residual network architecture can enhance classification performance on known classes, it may not necessarily improve open-set recognition capabilities.

On the DSIAC dataset, using the Classifier32 network, our method achieved a classification accuracy of 82.86% and an AUC of 0.8974. When using the ResNet-18 network, the classification accuracy improved slightly to 83.84%, but the AUC decreased to 0.8220. Similar to the observations on CIFAR-10, the residual network architecture improved classification accuracy but had a reduced AUC.

**Figure 5:**
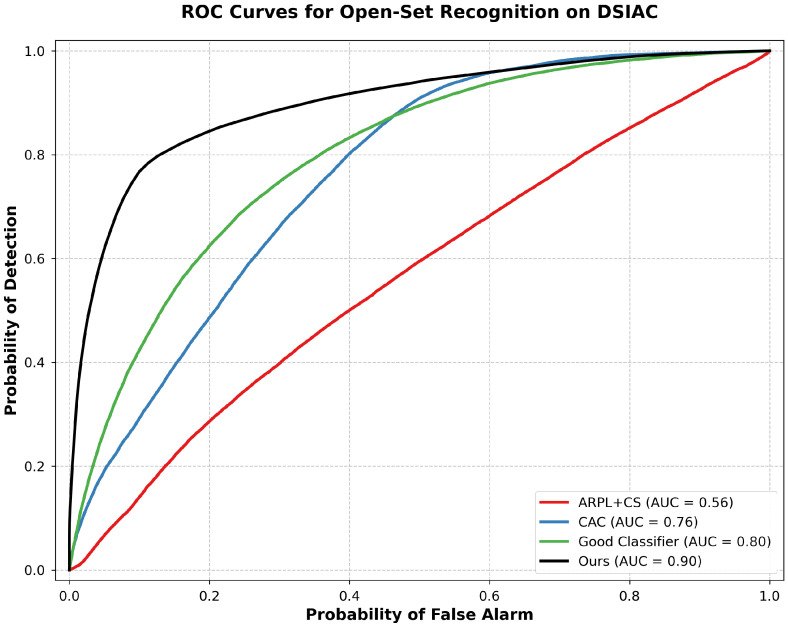
ROC curves for open-set recognition on the DSIAC dataset comparing our approach with ARPL + CS, CAC, and Good Classifier.

Figure 5 illustrates the ROC curves for different methods on the initial DSIAC experiment using the Classifier32 network. Our approach demonstrates a superior trade-off between the true positive rate and the false positive rate compared to existing methods.

Figure 3 shows the ROC curves for the DSIAC experiment where tracked vehicles are the known classes and wheeled vehicles are the unknowns. Our method achieves an AUC of 0.79 and an accuracy of 78.66%. The baseline methods ARPL + CS, CAC, and Good Classifier achieve AUCs of 0.61, 0.67 and 0.53 with accuracies of 27.78%, 76.06%, and 78.26% respectively.

Figure 4 shows the ROC curves for the DSIAC experiment where wheeled vehicles are the known classes and tracked vehicles are the unknowns. Our method achieves an AUC of 0.87 and an accuracy of 69.94%. The baseline methods ARPL + CS, CAC, and Good Classifier achieve AUCs of 0.45, 0.53, and 0.80 with accuracies of 55.05%, 79.58%, and 75.86% respectively.

## 5. Conclusions

This work introduces a novel approach for enhancing the ability of existing (or pre-trained) classifiers to reject images of unknown classes. Essentially, we combine the decision confidence of a conventional classifier with a class-conditional score produced by a regression model. By integrating class probabilities with normalized detection scores, the proposed method enhances the ability to distinguish between known and unknown inputs. This dual-model framework enables more informed decisions on unseen data, resulting in reliable performance in challenging contexts.

The broader implications of this research are significant, particularly in environments where the capability to handle unknown inputs is critical. In applications such as military systems, reliably identifying and managing unknown data enhances operational safety and reliability in unpredictable environments. Our approach enables enhanced the OOD rejection capability without compromising the recognition of known classes.

Future research will explore alternative methods for thresholding and score normalization to further enhance the discrimination between known and unknown inputs. Additionally, optimizing the integration of classification and detection components could help minimize computational resources, making the framework more suitable for deployment in resource-constrained settings. These directions hold promise for advancing the efficiency and effectiveness of open-set recognition techniques.

## Figures and Tables

**Table 1:** Performance comparison on the CIFAR-10 and DSIAC datasets. Bold values indicate the best performance for each metric within the dataset.

	CIFAR-10		DSIAC	
Method	Acc. (%)	AUC	Acc. (%)	AUC
ARPL + CS	78.13	0.7813	57.56	0.5600
CAC	71.56	0.7156	81.79	0.7600
Good Classifier	74.79	0.7479	79.90	0.7961
Ours (Classifier32)	88.18	**0.8654**	82.86	**0.8974**
Ours (ResNet-18)	**92.47**	0.8294	**83.84**	0.8220

## Data Availability

The datasets analyzed during the current study are available in the following repositories: CIFAR-10 is available at https://www.cs.toronto.edu/~kriz/cifar.html (accessed on 1 March 2024); DSIAC dataset must requested at https://dsiac.dtic.mil/ (accessed on 1 August 2020).

## Abbreviations

The following abbreviations are used in this manuscript:

ATR	Automatic target recognition
MWIR	Medium-wave infra-red
OOD	Out of distribution
ROC	Receiver operating characteristic
AUC	Area under the curve
CAC	Class anchor clustering
ARPL	Adversarial reciprocal points learning
CS	Confusing samples
MSP	Maximum softmax probability
